# Cataract surgery under topical anesthesia using 2% lignocaine jelly and intracameral lignocaine: Is manual small incision cataract surgery comparable to clear corneal phacoemulsification?

**DOI:** 10.4103/0301-4738.71713

**Published:** 2010

**Authors:** Sanjiv K Gupta, Ajai Kumar, Swati Agarwal

**Affiliations:** Ophthalmology Department, Chhatrapati Sahuji Maharaj Medical University, India; 1 DOMS, Jan Kalyan Eye Hospital, Lucknow, India

**Keywords:** Manual small incision cataract surgery, phacoemulsification, topical anesthesia, Visual analog scale, pain evaluation, gender, cataract. intracameral lignocaine

## Abstract

A prospective comparative study was undertaken to compare the patients’ pain experience, surgical outcome and surgeon’s experience in phacoemulsification and manual small incision cataract surgery (MSICS) under topical anesthesia supplemented with intracameral lignocaine (TASIL). In Group 1 (n=88) phacoemulsification was done and in Group 2 (n=92) MSICS was done. Pain scores were marked by the patients on a Visual analog scale (VAS) after the surgery. The surgical experience was noted on a questionnaire by the operating surgeon. Descriptive analysis and one-tailed Mann-Whitney test were used to draw results. The average VAS score in Group 1 was 0.65 (SD 1.31) and in Group 2 it was 0.90 (SD 1.22). This difference in the average was not statistically significant with *P*=0.09. The study demonstrates that MSICS and phacoemulsification both can be done safely under TASIL with acceptable patient comfort, and the pain experienced by the patients during the procedures is comparable.

Phacoemulsification is largely performed under topical anesthesia and manual small incision cataract surgery (MSICS) is done under local injectable anesthesia.

MSICS has been performed under topical anesthesia supplemented with intracameral lignocaine (TASIL) with acceptable patient comfort.[1] Comparative evaluation of phacoemulsification with MSICS under TASIL can provide us with conclusive evidence regarding the effectiveness of TASIL for MSICS.

The primary aim of the study was to evaluate and compare the pain in clear corneal phacoemulsification with foldable posterior chamber intraocular lens (PCIOL) to pain during MSICS using a sclero-corneal tunnel with rigid PCIOL done under TASIL. Secondary aims were to compare the surgeon’s experience during the two methods of cataract surgery and to evaluate the surgical outcome and complications of the two groups.

## Materials and Methods

This was a prospective nonrandomized comparative study to compare the patients’ experience, surgeon’s experience and the outcome of cataract surgery under TASIL done either by phacoemulsification or MSICS.

Subjects were the patients who underwent cataract surgery with intraocular lens (IOL) for visual rehabilitation for significant cataract at our eye hospital.

Patients with history of any previous intraocular surgery, sensitivity to lignocaine and uncooperative attitude (children, mentally challenged, involuntary movements) were excluded from the study.

**Figure 1:**
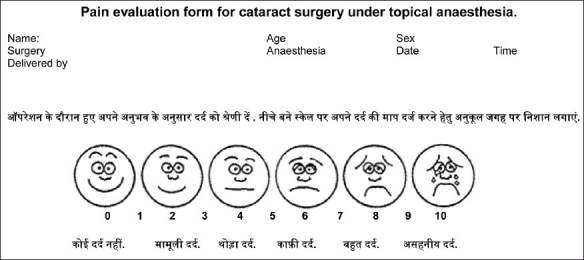
Pain evaluation form for patients undergoing cataract surgery under topical anesthesia

Patients were recruited in the two groups by their own choice, largely guided by the difference in the cost of the surgery (phacoemulsification with foldable IOL being expensive as compared to MSICS with rigid IOL). No blinding could be adopted due to standard consent procedures. Patients were instructed to inform verbally about any discomfort or pain during the surgery. At the end of the surgery the eye was patched and the patients were given a modified visual analog scale (VAS)[1] to report their pain experience by marking at the appropriate point. For those patients who could not comprehend the scale, there was an integrated modified Wong scale in the local language [Fig. 1].

**Figure 2:**
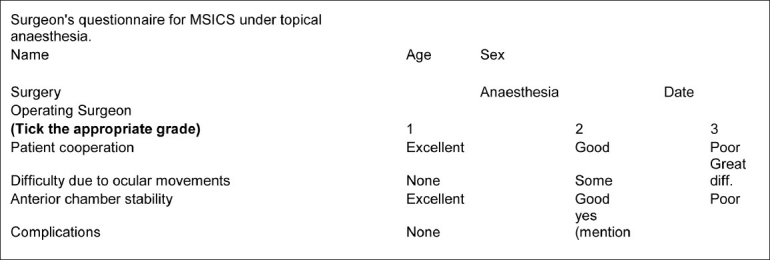
The surgeon’s evaluation form for cataract surgery under topical anesthesia

Single surgeon performed the surgeries in both the groups. The surgeon also ranked his experience of the surgery by a questionnaire. The questionnaire was designed in a manner so that the lower score would indicate a more cooperative patient and comfortable surgery. The score could range from 3 to 9 [Fig. 2].

The eye patch on both the groups was removed after half an hour and steroid and antibiotic eye drops were initiated after obtaining their VAS score.

We arrived at the sample size of 88 by using the following parameters, confidence level 95%, confidence interval of 10, and the total population under question 1000 (the total number of eyes undergoing cataract surgery at the hospital). Anticipating that the pain evaluation scores will follow a non-Gaussian distribution, we used Mann-Whitney test to compare the two groups. Descriptive statistics were used to comment upon the other parameters and their distribution.

### Surgical technique

Preoperative pupillary dilatation was achieved by tropicamide 0.8% and phenylephrine 5% eye drops and flurbiprofen 0.03% eye drops. Lignocaine drops 2% were instilled in the conjunctiva sac in the preoperative room 10 min before the onset of surgery. After draping the patient eye speculum was inserted and a copious amount of lignocaine 2% jelly (Xylocaine jelly 2% Astra Zeneca)[1] was poured on the exposed ocular surface to cover it. Subsequently the steps were different in the groups and are described separately

Group 1: After waiting for about a minute, clear corneal phacoemulsification using direct horizontal chop technique was done and foldable 6 mm optics, hydrophilic acrylic IOL (Ocuflex^TM^ Lens Style ANU 6 by the Care Group) was implanted. Intracameral 0.5% lignocaine (preservative-free) was used to supplement topical anesthesia (TA). The wound was checked for leakage and edges hydrated if needed. Eye was patched after the completion of the surgery.

Group 2: MSICS under TASIL was done as described by Gupta *et al*.,[1] A rigid Poly Methyl MethAcrylate (PMMA) 6.5 mm optics PC IOL (Preziol by Care Group) was implanted in the bag under 2% methylcellulose. At the end 0.25 ml subconjunctival injection of antibiotic steroid solution was given, which caused ballooning of conjunctiva and helped in repositioning the conjunctiva. The eye was patched after the completion of the surgery.

## Results

The study included 180 eyes of 180 patients. Eighty-eight patients were in Group 1 (phacoemulsification) and 92 patients in Group 2 (MSICS). There were 93 females (Group 1 n=50, Group 2 n=43) and 87 males (Group 1 n=38, Group 2 n=49).

**Figure 3:**
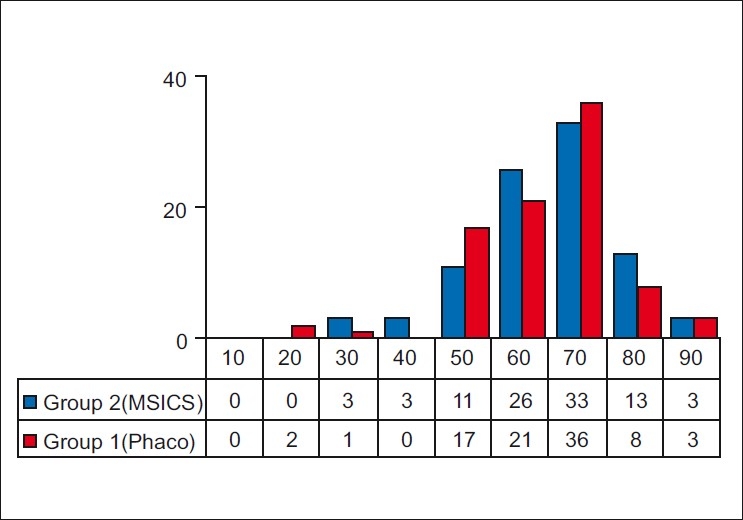
Age distribution histogram in the two groups

Age distribution is presented in Fig. 3. The mean age in the groups was 60.13 years (SD 12.32, range 14-85 years) in Group 1 and 60.82 years (SD 12.19, range 21-87 years) in Group 2. This difference in the mean age was not significant (*P*=0.70, student t test, two-tailed assuming equal variances).

**Figure 4:**
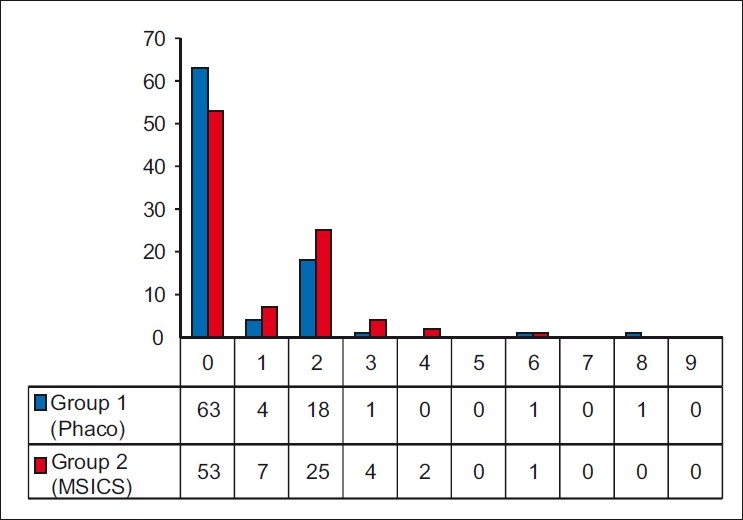
Visual analog scale score for pain during cataract surgery under topical anesthesia

The pain evaluation score is depicted in the histogram [Fig. 4]. The average VAS score in Group 1 was 0.65 (SD 1.31, range 0-8) and in Group 2 it was 0.90 (SD 1.22, range 0-6). This difference in the average was not statistically significant with *P*=0.09 (Mann-Whitney test, one-tailed presuming VAS score in Group 1 to be less than Group 2). Seventy per cent subjects in Group 1 and 57% patients in Group 2 had a score of zero, which is no pain.

**Figure 5:**
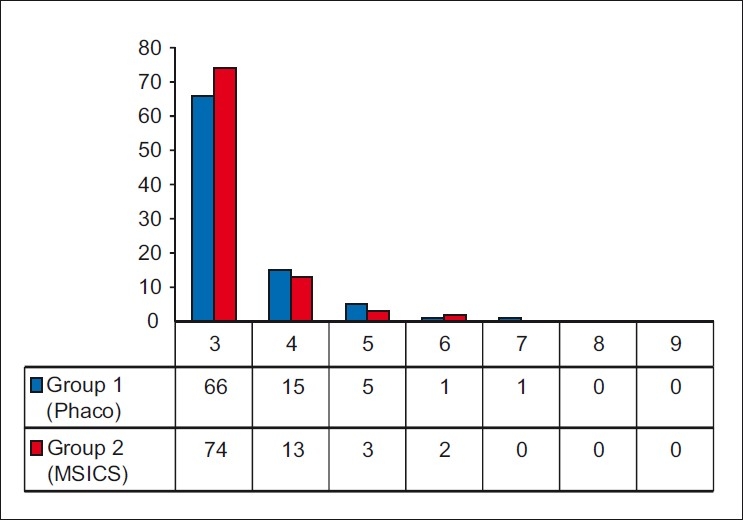
Histogram of surgeon’s score for surgery under topical anesthesia

There were no surgical complications which could compromise the visual outcome and only minor complications were seen unrelated to the anesthesia technique [Table 1]. The frequency distribution of the surgeon’s score for the two groups is shown in Fig. 5. The outcome showed that the average score in Group 1 (phacoemulsification) was 3.3 (SD 0.7, range 3-7) and in Group 2 it was 3.27 (SD 0.63, range 3-6) (Lower score indicates a more favorable experience). The difference between the means was insignificant with a P value of 0.18 (Mann-Whitney one-tailed test).

**Table 1:** Incidence of complications during surgery under topical anesthesia

Complications in cataract surgery under topical anesthesia	Group 1 (Phacoemulsification)	Group 2 (MSICS)
Excessive Blepharospasm	3 (3.4)	4 (4.3)
Excessive eye movements	2 (2.2)	3 (3.2)
Bleeding	0	5 (5.4)
Conjunctival chemosis	4 (4.5)	0
Radial keratotomy scar Dehiscence	1 (1.1)	0
Iris trauma	0	2 (2.1)
Incomplete rhexis	2 (2.2)	4 (4.3)
Posterior capsular tear	0	0
Anterior chamber leakage (managed by air bubble)	3 (3.4)	0
Descetmets’ detachment	0 2	(2.1)

Figures in parentheses are in percentage

There was no sight-threatening complication in any patient during surgery. Blepharospasm and unwanted eye movements are integral difficulties faced by the surgeons during TA. In our series we had a few patients (~5% and ~7% in Group 1 and Group 2 respectively), however there was no need to convert to local anesthesia. Bleeding from the sclera blood vessels (~5%) and descemet’s detachments (~2%) were the complications limited to Group 2. On the other hand conjunctival chemosis (4.5%) and anterior chamber leakage (3.4%) managed by air injection in the anterior chamber was seen exclusively in Group 1 patients. One patient in Group 1 had dehiscence of the Radial Keratotomy scar (done 12 years back) which was managed by a single suture of 10-0 nylon. None of the complications were sight-threatening and were unrelated to the anesthesia technique.

## Discussion

TASIL may be used for MSICS as well, retaining all the mentioned advantages with acceptable patient comfort and successful surgery.[2]

Our VAS score of 0.65 (SD 1.31) in Group 1 is lower than the reports from other studies on phacoemulsification under topical anesthesia with intracameral lignocaine, (similar to Group 1) who have reported the average VAS scores as 0.92 (SD1.34),[3] 2.02 (SD2.35),[4] 1.13 (SD 1.36) and 0.84 (SD 1.30).[2] This is because we have used lignocaine viscous instead of drops, which is known to provide comparative[5,6] and even better anesthesia for cataract surgery[7,8] when compared to anesthetic drop preparations. The VAS score in Group 2 was similar to a study on MSICS under TASIL (similar to Group 2) which had shown a VAS score of 0.70 (SD ±0.97).[1]

The study has a few limitations. Being a non-randomized study it has less weightage. The study was done at a single centre and by a single surgeon, thus omitting the differences due to surgical techniques and skill. A more widespread use of TASIL and future comparative studies may further highlight the acceptability of TASIL for MSICS.

## Conclusions

The study results demonstrate that both phacoemulsification and MSICS can be done under TASIL with acceptable patient comfort and, the pain experienced in both the techniques is comparable. Further, the study also highlights that the TASIL is comfortable for the operating surgeon and does not compromise surgical results when used for MSICS.
